# Supplementary material for: Exploring the consequences of redirecting an exocytic Rab onto endocytic vesicles
Source: bioRxiv. 2023 Feb 10:2023.02.09.527811. Preprint. [Version 1] doi: 10.1101/2023.02.09.527811 (PMC9934678; doi:10.1101/2023.02.09.527811)
Supplement: Supplement 1 [file NIHPP2023.02.09.527811v1-supplement-1.pdf]

bioRxiv preprint doi: https://doi.org/10.1101/2023.02.09.527811; this version posted February 10, 2023. The copyright holder for this preprint (which was not certified by peer review) is the author/funder, who has granted bioRxiv a license to display the preprint in perpetuity. It is made available under aCC-BY 4.0 International license.

Figure S1. Vps9-mCherry partially colocalizes with Sec2GEF-GFP-CUE but not with Sec2-GFP. Representative images of Sec2-GFP or Sec2GEF-GFP-CUE, Vps9-mCherry, merged and overlay DIC with merged images are shown on left panel. Closed arrowheads indicate GFP puncta with which Vps9-mCherry is not colocalized. Open arrowheads and arrows point to GFP puncta colocalized with Vps9-mCherry at polarized sites and non-polarized sites, respectively. The localization of Sec2GEF-GFP-CUE or Sec2-GFP and their colocalization with Vps9-mCherry were quantified and shown on the right panel. The error bars represent SD from three independent experiments. Bar, 5 µm.

Figure S2. Localization of Sec2GEF-GFP-CUE (Sec2GEF-mCherry-CUE) or Sec2-GFP (Sec2-mCherry) and their colocalization with Sec4, Sec8 or Sec7 in *vps4*Δ strains. **(A).** Confocal fluorescence micrographs of Sec2-GFP or Sec2GEF-GFP-CUE in *vps4*Δ strains. Left panel shows representative GFP fluorescence, DIC and merged images of Sec2-GFP or Sec2GEF-GFP-CUE in *vps4*Δ background, and the quantitation results of GFP puncta localized at polarized sites (open arrowheads) or non-polarized sites (arrows) are shown on right panel. **(B).** Live-cell fluorescence microscopy of *SEC2-mCherry* and *SEC2GEF-mCherry-CUE* strains expressing GFP-Sec4 in *vps4*Δ strains. Left panel shows representative GFP fluorescence, mCherry fluorescence, merged, and DIC overlaid with merged images. The quantification of Sec2-mCherry and Sec2GEF-mCherry-CUE localization at polarized or non-polarized sites and their colocalization with GFP-Sec4 protein are shown on the right panel. Open arrowheads and arrows indicate respectively polarized sites and non-polarized sites at which mCherry colocalizes with GFP-Sec4 protein. **(C-D).** Upper panels show representative images of GFP channel, Sec8-mCherry (C) or Sec7-dsRed (D), merged channels and overlay DIC with merged images in

bioRxiv preprint doi: https://doi.org/10.1101/2023.02.09.527811; this version posted February 10, 2023. The copyright holder for this preprint (which was not certified by peer review) is the author/funder, who has granted bioRxiv a license to display the preprint in perpetuity. It is made available under aCC-BY 4.0 International license.

SEC2-GFP or *SEC2GEF-GFP-CUE* strains. Open arrowheads indicate polarized sites at which GFP colocalizes with dsRed or mCherry-tagged proteins, while closed arrowheads point to GFP puncta which are not colocalized with the proteins examined. The quantification of Sec2-GFP and Sec2GEF-GFP-CUE colocalization with Sec8-mCherry or Sec7-dsRed are shown on lower panels. In all quantitation graphs the error bars represent the SD from three independent experiments. Scale bar, 5 µm.

Figure S3. Colocalization of Sec2GEF-GFP-CUE or Sec2-GFP with mCherry-FAPP1-PH or mRFP-FYVE(EEA1) in *wild type VPS4* (A and C) or *vps4*Δ strains (B and D). (**A) and (B).** Shown on upper panels are representative Sec2-GFP or Sec2GEF-GFP-CUE, mCherry-FAPP1-PH, merged and overlay DIC with merged images. Closed arrowheads point to Sec2-GFP or Sec2GEF-GFP-CUE not colocalizated with mCherry-FAPP1-PH. There is very low percentage of colocalization of mCherry-FAPP1-PH with Sec2GEF-mCherry-CUE in *vps4*Δ strains. The quantification results are shown on the lower panels. **(C) and (D).** Shown on top panels are representative images of the mRFP channel, GFP channel, merged channels and overlay DIC with merged channels. Open arrowheads or closed arrowheads point to respectively colocalized or non-colocalized Sec2-GFP or Sec2GEF-GFP-CUE with mRFP-FYVE(EEA1). There is a significant amount of colocalization of mRFP-FYVE(EEA1) with Sec2GEF-GFP-CUE in *vps4*Δ strains at both polarized and non-polarized sites, but no colocalization of mRFP-FYVE(EEA1) with Sec2-GFP in either strain. The quantification results are shown on bottom panels. The error bars represent SD from three independent experiments. Scale bar, 5 µm.

Figure S4. Colocalization of Sec2GEF-GFP-CUE, Sec2GEF-mCherry-CUE, Sec2-GFP or Sec2-

bioRxiv preprint doi: https://doi.org/10.1101/2023.02.09.527811; this version posted February 10, 2023. The copyright holder for this preprint (which was not certified by peer review) is the author/funder, who has granted bioRxiv a license to display the preprint in perpetuity. It is made available under aCC-BY 4.0 International license.

mCherry with Sec15-mCherry, GFP-Sec9, Sec3-mCherry or Exo70-GFP. (**A**)**.** Shown on upper panel are representative Sec2-GFP or Sec2GEF-GFP-CUE, Sec15-mCherry, merged and overlay DIC with merged images. Open arrowheads point to Sec15-mCherry colocalization with Sec2-GFP or Sec2GEF-GFP-CUE at polarized sites. The quantification results are shown on the lower panel. (**B**). GFP-Sec9 expressed from the chromosomal locus exhibits little co-localization with Sec2GEF-mCherry-CUE. Upper panel shows representative mCherry-labeled Sec2 or Sec2GEF-CUE, GFP-Sec9, merged and overlay DIC with merged images. Open arrowheads point to its colocalization with Sec2-mCherry or Sec2GEF-mCherry-CUE at polarized sites, while closed arrowheads point to non-colocalized GFP-Sec9 with Sec2GEF-mCherry-CUE. There is a low percentage of colocalization of GFP-Sec9 with Sec2GEF-mCherry-CUE at polarized sites and barely any colocalization with Sec2-mCherry or Sec2GEF-mCherry-CUE at non-polarized sites. The quantification results are shown on lower panels. **(C) and (D).** Shown on top panels are representative images of the mCherry channel, GFP channel, merged channels and overlay DIC with merged channels. Open arrowheads or closed arrowheads point to respectively colocalized or non-colocalized exocyst Sec3 or Exo70 with Sec2 or Sec2GEF-CUE at polarized sites. There is little colocalization of exocyst Sec3 or Exo70 with Sec2 or Sec2GEF-CUE at non-polarized sites. The quantification results are shown on the middle panels. Among the Sec3 or Exo70 structures that fail to colocalize with Sec2GEF-CUE at polarized sites, most are directly adjacent to a Sec2GEF-CUE structure. The percentage of cells containing Sec2GEF-CUE or Sec2 at polarized sites among which Sec3 or Exo70 is either colocalized or adjacent were quantified and shown on bottom panels. The error bars represent SD from three independent experiments. Scale bar, 5 μm.

bioRxiv preprint doi: https://doi.org/10.1101/2023.02.09.527811; this version posted February 10, 2023. The copyright holder for this preprint (which was not certified by peer review) is the author/funder, who has granted bioRxiv a license to display the preprint in perpetuity. It is made available under aCC-BY 4.0 International license.

Figure S5. Sec2GEF expressed from the Sec2 promoter is not sufficient to support cell growth while GFP tag and other tags confers stability of the fusion proteins. (**A**)**.** Protein expression level from the GFP-, mCherry- and NeonGreen- tagged Sec2 or Sec2GEF-CUE constructs under ADH1 promoter have been examined by anti-Sec2 western blot. The yeast lysates were extracted from strains NY3381, NY3384, NY3388, NY3390, NY3391 and NY3392, separately. Pgk1 was visualized as a loading control. (**B**)**.** Tetrad dissection analysis for diploid cells with one chromosomal *SEC2* copy replaced with the *HIS3* module and Sec2GEF expressed from the Sec2 promoter integrated at the *ura3* locus. After dissection, replica plating to infer genotype showed that the inviable spores were *HIS3* and were either *URA3* or *ura3*. (**C**). Yeast lysates were extracted separately from strains *wild type* (NY1210), *sec2Δ ura3-52::pSec2-SEC2GEF-GFP-CUE* (NY3385), *sec2Δ ura3-52::pSec2-SEC2GEF-GFP-CUE*^{M419D} (NY3386), *sec2Δ ura3-52::pSec2-SEC2GEF-GFP* (NY3387) or *SEC2 ura3-52::pSec2-SEC2GEF* (NY3445). Sec2 protein level was measured using anti-Sec2 western blot.

Figure S6. *SEC2GEF-CUE* cells do not exhibit significant secretion defects by Bgl2 secretion assay. (**A**). Strains *WT*, *SEC2GEF-GFP-CUE, SEC2GEF-GFP-CUE*^{M419D}*, SEC2GEF-GFP, SEC2-GFP* or secretion mutant strain *sec6-4* were grown at 25^{0}C in YPD overnight to early-log phase and shifted to 37^{0}C for 1hr before harvesting. Internal and external fractions of Bgl2 glucanase were prepared as described in Materials and Methods. Eight-fold more of the internal fraction was loaded relative to the external fraction. Bgl2 was visualized by western blotting using anti-Bgl2 rabbit polyclonal antibody. (**B**). Normalized quantitation of internal Bgl2 percentage from the blots on panel **A** is shown. Error bars represent SD from three independent experiments.

bioRxiv preprint doi: https://doi.org/10.1101/2023.02.09.527811; this version posted February 10, 2023. The copyright holder for this preprint (which was not certified by peer review) is the author/funder, who has granted bioRxiv a license to display the preprint in perpetuity. It is made available under aCC-BY 4.0 International license.

Figure S7. The *SEC2GEF-CUE* mutant has very mild secretion defects alone, yet the defects are more severe in *sro7Δ* cells. (**A).** Strains WT, *sro7Δ, sro7Δ SEC2GEF-GFP-CUE, sro7Δ SEC2-GFP* were grown at 25^{0}C in YPD overnight to mid-log phase. Internal and external fractions of Bgl2 glucanase were isolated and analyzed as described in Materials and Methods. Ten-fold more of the internal fraction was loaded relative to the external fraction. Bgl2 was visualized by western blotting using anti-Bgl2 rabbit polyclonal antibody. Normalized quantitation of internal Bgl2 percentage from the blots on the left panel is shown on the right panel. Error bars represent SD, n=3. ns, not significant, * P< 0.05, ** P< 0.01. (**B-C)**. Thin-section EM images from the strains indicated are prepared as described in Materials and Methods. The cells were fixed in cacodylate/glutaraldyhyde and stained with lead citrate/uranyl acetate. Representative images at 10000x magnification are shown. Arrowheads indicate vesicles and Scale bars are shown as indicated. Quantitation of secretory vesicles per cell are shown in panel C. Over 100 cells were analyzed for each strain.

Figure S8. Endocytosis followed by Mup1-GFP delivery to the vacuole for degradation is slow in *SEC2GEF-mCherry-CUE* cells. (**A**). Live-cell fluorescence microscopy of Mup1-GFP in *Sec2GEF-mCherry-CUE* (upper panel) and *Sec2-mCherry* cells (lower panel) after the addition of 20μg/ml methionine to stimulate internalization of Mup1-GFP from the plasma membrane and transport to the vacuole lumen. Representative images at indicated time point have been displayed and GFP fluorescence patches in the vacuole were indicated by arrowheads. Scale bar, 2 μm. (**B**). The percentage of cells containing GFP fluorescence in the vacuole at two time points 63min and 93min after the addition of methionine was quantified and plotted. Error bars

bioRxiv preprint doi: https://doi.org/10.1101/2023.02.09.527811; this version posted February 10, 2023. The copyright holder for this preprint (which was not certified by peer review) is the author/funder, who has granted bioRxiv a license to display the preprint in perpetuity. It is made available under aCC-BY 4.0 International license.

represent SD from three independent experiments.

Movies S1 and S2. Vesicle dynamics as seen in Sec2GEF-Neongreen-CUE labeled cells (S1) and in wild-type Sec2-Neongreen labeled cells (S2). The acquisition was taken using SORA super resolution setting with 40% 488nm laser power and images are processed using AI enhancement. Acquisition time duration is 30sec without interval delay; movie speed, 10 frames per second = 1.7s/s. One z-axis plane fluorescence image was acquired.

Movies S3 and S4. Examples of Fluorescence Recovery After Photobleach (FRAP) in entire small buds of *SEC2GEF-Neongreen-CUE* cells (S3) and wild-type *SEC2-Neongreen* cells (S4). Acquisition was taken using regular setting with 40% 488nm laser power. Photobleaching of the entire bud was performed using 10% laser power at 405nm with a 1-ms dwell time and a raster block size of 10 (duration 100ms/point). Acquisition time duration is 30sec without interval delay; movie speed, 10 frames per second = 2s/s. One z-axis plane fluorescence image was acquired.

Movies S5 and S6. Show examples of bleached cells used in vesicle tracking studies for wild-type *SEC2-Neongreen* (S5) and *SEC2GEF-Neongreen-CUE* (S6) strains. Acquisition was taken using SORA super resolution setting with 20% 488nm laser power and images were processed using AI enhancement. Photobleaching of the entire bud was performed using 10% laser power at 405nm with a 1-ms dwell time and a raster block size of 10 (duration 100ms/point). Acquisition time duration is 1min with 1sec interval for *SEC2GEF-Neongreen-CUE* strain and 30sec with 1sec interval for *SEC2-Neongreen* strain since overall signal in *SEC2-Neongreen*

bioRxiv preprint doi: https://doi.org/10.1101/2023.02.09.527811; this version posted February 10, 2023. The copyright holder for this preprint (which was not certified by peer review) is the author/funder, who has granted bioRxiv a license to display the preprint in perpetuity. It is made available under aCC-BY 4.0 International license.

strain was weaker; movie speed, 10 frames per second = 10s/s. Five z-axis plane fluorescence images were acquired and merged.

Movies S7 and S8. Endocytosis of Mup1-GFP in *SEC2-mCherry* cells (S7) and in *SEC2GEF-mCherry-CUE* cells (S8). The acquisition was started 3min after 20µg/ml methionine addition. Acquisition time duration was 60min with 1min interval; movie speed, 2 frames per second = 2 min/s. One z-axis plane fluorescence image was acquired.

Table SI: Yeast strains.

Table SII: Bacterial plasmids.
